# Fast and reliable association discovery in large-scale microbiome studies and meta-analyses using PALM

**DOI:** 10.64898/2026.04.09.717497

**Published:** 2026-04-10

**Authors:** Zhoujingpeng Wei, Qilin Hong, Guanhua Chen, Tina V. Hartert, Christian Rosas-Salazar, Suman R. Das, Meghan H. Shilts, Albert M. Levin, Zheng-Zheng Tang

**Affiliations:** 1 Department of Biostatistics and Medical Informatics, University of Wisconsin-Madison, Madison, WI, USA; 2 Department of Medicine, Vanderbilt University Medical Center, Nashville, TN, USA; 3 Department of Pediatrics, Vanderbilt University Medical Center, Nashville, TN, USA; 4 Department of Public Health Sciences, Henry Ford Health System, Detroit, MI, USA; 5 Twitter handle: @tangzz_lab

**Keywords:** absolute abundance, compositional data, differential abundance, microbiome meta-analysis, quasi-Poisson, relative abundance, score statistics, summary statistics

## Abstract

Identifying microbial features associated with various covariates is a long-standing goal in microbiome research. Modern association studies incorporate an ever-increasing number of microbial features, covariates, and datasets from diverse cohorts. However, the complexity of microbiome data challenges analysis, often leading to poor replication of findings. We introduce PALM, a quasi-Poisson regression framework that enables fast and reliable association discovery in large-scale studies and meta-analyses. Extensive, realistic simulations demonstrate PALM’s advantages in controlling false discovery rates, boosting power, improving computational efficiency, and preserving cross-study homogeneity of association effects. Three real-world applications at different scales illustrate PALM’s utility, underscoring its potential to advance microbiome research.

## Background

Significant progress has been made in understanding the role of the human microbiome in health and disease^1,2^. Feature-level association analyses, commonly known as differential abundance analyses, are widely used to identify microbial features associated with clinical outcomes, host factors, and other relevant covariates. Recent advances have expanded microbial feature measurements beyond 16S rRNA gene amplicon profiling to multi-omics approaches, including metagenomics and metatranscriptomics^3,4^. Additionally, microbiome studies increasingly link microbial features to an ever-growing set of covariates^5,6^. For example, microbiome genome-wide association studies (mbGWAS) link microbial features to millions of host genetic variants. Furthermore, meta-analyses of microbiome association studies are becoming more common, aiming to enhance discovery power and identify microbial signatures that are generalizable across cohorts and populations^7–10^. The substantial growth in study scale offers an unprecedented opportunity to uncover novel microbial signatures, deepen our understanding of microbiome functions, and translate these insights into actionable interventions. However, poor replication of findings remains a significant barrier to achieving these goals. For instance, while existing mbGWAS have identified numerous genetic loci associated with microbial taxa, only a handful of the associations were shared across studies^11^.

Poor replication is due in part to the analytical challenges posed by the unique characteristics of microbiome data. Microbiome data consist of sequencing read counts mapped to microbial features. Because sequencing depth is uncorrelated with the actual microbial load in a sample, these counts are inherently compositional and are informative about relative abundances (RA) but not about absolute abundances (AA) per unit volume of the ecosystem^12,13^. These count data are inherently noisy. Measurement biases are introduced during experimental workflows (e.g., DNA extraction, primer binding, and amplification), leading to differential efficiencies in detecting features^14^. The majority of counts are concentrated in a few abundant features, with the remainder distributed among numerous low-abundance features. This results in a high proportion of zero counts, with the likelihood of observing zeros heavily influenced by a sample’s sequencing depth. Zero counts can also arise from true feature absence, which is difficult to distinguish from zeros due to insufficient sampling. The counts also exhibit substantial variation across samples, particularly when generated from different sequencing batches, study designs, or populations. These unique characteristics make microbiome data difficult to model.

Despite extensive research on microbiome association analysis, existing methods still struggle with inflated false discovery rates (FDR) and unstable effect estimation. In meta-analyses, two distinct forms of heterogeneity must be disentangled. Effect heterogeneity refers to genuine differences in the association parameter across studies, whereas distributional heterogeneity (batch effects) arises when observed microbiome profiles differ across studies because of technical or cohort factors (e.g., extraction protocols, sequencing platforms, or population structure)^9,15,16^. Batch effects alter the data distribution but do not, by themselves, imply that the underlying biological effects differ across studies. However, if study-level analyses are not properly specified, batch structure can propagate into the estimated effects, making truly homogeneous effects appear heterogeneous.

Connecting back to the unique characteristics of microbiome data, this problem can arise from several sources: (1) Data preprocessing. Many methods^5,17–19^ rely on normalization (sample-level scaling or transformations) and zero imputation; in meta-analyses, cross-study batch correction is also common^16^. These steps can distort target associations and propagate batch structure into association effects^20–23^. (2) Compositionality. Many approaches^5,17^ target RA associations. Changes in one feature’s AA alter all RAs. Consequently, methods that ignore compositional effects can yield biased estimates of AA-level associations and propagate batch-induced shifts across features. Compositionality-aware methods^18,19,24^ improve FDR control but have not been comprehensively evaluated in meta-analysis settings. (3) Modeling and inference. Misspecified parametric models or unstable inference for sparse, over-dispersed counts can bias both effect estimates and their uncertainty quantification, leading to inflated FDR and apparent between-study effect heterogeneity in meta-analysis.

In this article, we introduce a semi-parametric quasi-Poisson method for Association analysis of Large-scale Microbiome studies and meta-analysis (PALM). PALM provides fast and reliable estimates of AA-level association effects with well-calibrated uncertainty quantification. In meta-analyses, PALM integrates the AA-level summary statistics across studies, preserving genuinely homogeneous effects and supporting reproducible, generalizable findings. We evaluate the performance of PALM through extensive realistic simulations for meta-analysis and illustrate its utility in three real data applications: identifying microbial features associated with colorectal cancer, hundreds of metabolites, and millions of host genetic variations. These studies show that PALM achieves superior FDR control, power, and speed and avoids spurious between-study heterogeneity in association effects.

## Results

### PALM overview

PALM is detailed in Methods, with a schematic overview for meta-analysis provided in Additional file 1: Fig. S1. In brief, PALM takes as input the microbiome count data, sequencing depth, the covariate of interest, and relevant confounders. For each study, PALM generates summary statistics for individual features, including the AA-level association effect estimates and their variances. These summary statistics are subsequently combined across studies to perform a meta-analysis and calculate overall association test *p*-values. The *p*-values are then adjusted for multiple testing over features, and a list of significant features exceeding the specified threshold is reported.

The modeling and estimation procedure of PALM offers a unique combination of advantages: (i) No data preprocessing requirements: PALM directly models count data, avoiding biases and instability introduced by data normalization, zero imputation, and batch effect correction. (ii) No distributional assumptions: PALM employs a semi-parametric approach that accommodates overdispersion in count data without relying on strict distributional assumptions. This results in more reliable inference compared to parametric models. (iii) Compositional effect correction: PALM connects association effects in the RA model to those in the latent AA model. Specifically, the RA-model intercept absorbs multiplicative nuisance from total microbial load and measurement bias, and RA effects equal AA effects minus a common, study-specific shift. By leveraging this relationship and the high dimensionality of features, PALM accurately corrects for compositional effects and recovers AA-level summary statistics. (iv) Fast and stable inference: PALM constructs summary statistics using score statistics, which improves numerical stability and accuracy for sparse and over-dispersed microbiome count data compared to Wald statistics. Importantly, these score statistics are computed by fitting a single null model independent of the covariate of interest, making PALM computationally efficient for association studies with large numbers of covariates. (v) Flexibility: PALM supports diverse study designs, such as cross-sectional and longitudinal studies, and allows for adjustment of confounders.

### Evaluations on realistic simulated data

We evaluated the performance of PALM and compared it with several leading methods in the meta-analysis of five studies testing for differential AA between two comparison groups. Each method generated summary statistics, which were combined using a fixed-effect meta-analysis, as the simulated association effects were homogeneous across studies. The compared methods included ANCOM-BC2^19^ and LinDA^18^, designed to detect differential AA. For ANCOM-BC2, we used the version that applies a feature-level sensitivity score filter to reduce potential false positives^19^. Therefore, only studies where a feature passed the sensitivity score contributed to the ANCOM-BC2 meta-analysis for that feature. Additionally, we included DESeq2^17^, MaAsLin2^5^, and LM-CLR^25^, which are widely used for detecting differential RA. LM-CLR is a linear model that uses the centered log-ratio (CLR)-transformed feature variable as the response.

We simulated microbiome data for five studies using five real metagenomics datasets as templates^8^. The real data exhibited systematic differences in the structures of microbial communities across studies (Additional file 1: Fig. S2a). To generate realistic simulated data, we used the microbiome data simulator MIDAsim^26^, which learned the structure of the template data for each study and produced data closely resembling the originals (Additional file 1: Fig. S3). Due to the information gap in realistically generating AA data from RA data, we directly introduced differential AA signals into the RA data and ensured the effects were homogeneous across studies (Methods). We evaluated a broad range of scenarios from the combinations of two sample size settings (n=100,120,140,160,180 or n=20,40,60,80,100), two feature size settings (K=401 species or K=92 genera), two association effect direction settings (balanced positive/negative effects or predominantly positive effects), and two sequencing depth settings (even or uneven depths between groups of comparison). For each scenario, we also varied the proportion of AA-differential features, ranging from 0.05 to 0.2.

**Fig. 1:**
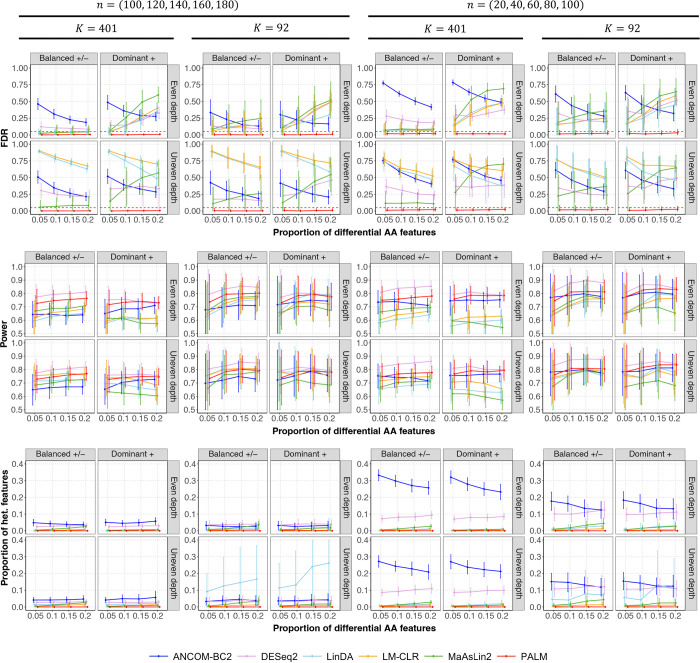
Comparison of different methods in the meta-analysis of five simulated studies with independent samples. The figure is divided into three sections, each representing a benchmark metric (y-axis): empirical FDR (top; the horizontal line indicates the target FDR), power (middle), and the proportion of heterogeneous features (bottom). Heterogeneous features are defined as those with variation in effect sizes across studies exceeding what is expected by chance (Cochran’s Q test q-value < 0.1). The x-axis represents the proportion of differential absolute abundance (AA) features. The top title shows the two scenarios of sample sizes n in five studies. The second title shows the two scenarios of feature size K. The column facet title represents two effect direction scenarios: Balanced +/−, where differential AA features have an equal probability of exhibiting positive or negative effects, and Dominant +, where all differential AA features exhibit positive effects. The row facet title represents two sequencing depth scenarios: one with even sequencing depth and one with uneven sequencing depth between the comparison groups. Each panel displays the mean estimated metrics with ± standard errors (indicated by error bars) based on 100 simulation replicates.

We evaluated all methods in terms of FDR control and discovery power (Fig. 1). For all methods, we applied the Benjamini-Hochberg (BH) procedure^27^ for multiple testing correction and reported empirical FDR and power at a target FDR of 0.05. PALM was the only method that successfully controlled FDR across all scenarios. Unbalanced positive/negative effects and uneven sequencing depth exacerbated FDR inflation in the compared methods. PALM consistently maintained high power, even when the other methods exhibited FDR inflation. PALM exhibits the best power–FDR trade-off, as reflected by the area under the precision–recall curve (AUPRC; Additional file 1: Fig. S4).

Evaluating effect size heterogeneity across studies is crucial in meta-analysis as it provides key insights into the consistency and generalizability of study findings. To this end, we reported the proportion of features with significant heterogeneity (Fig. 1), determined using Cochran’s Q test (q-val < 0.1). In our simulations, the true AA-level effects were homogeneous; therefore, accurate summary statistics should exhibit no heterogeneity. PALM summary statistics showed no heterogeneity, indicating that it preserves genuinely homogeneous effects without introducing spurious differences. Other methods exhibited varying degrees of effect heterogeneity, with ANCOM-BC2 summary statistics showing the highest level in most scenarios.

We particularly evaluated method performance for rare features. In our simulation design, half of the AA-differential features were randomly drawn from relatively abundant features (average proportion ≥ 10^−3^ in the template data), and the other half from rare features (average proportion < 10^−3^). In the Additional file 1: Fig. S5, we reported results restricted to the subset of rare features. PALM maintains FDR control with good power, whereas DESeq2 and ANCOM-BC2 exhibit more pronounced FDR inflation than in the all-feature evaluation.

We also compared PALM meta-analysis with pooled-data analyses by other methods (Additional file 1: Fig. S6). Specifically, the original individual-level data from all studies were pooled together, and study indicators were adjusted as confounders in the association analysis. For ANCOM-BC2, the pooled-data analysis achieved better FDR control compared to its meta-analysis, but this came at the cost of reduced power. This trade-off occurred because the ANCOM-BC2 sensitivity score filter removed both false positives and true positives in the pooled-data analysis. For DESeq2, LinDA, LM-CLR, and MaAsLin2, the FDR inflation in the pooled-data analysis was similar to that observed in their meta-analysis.

**Fig. 2:**
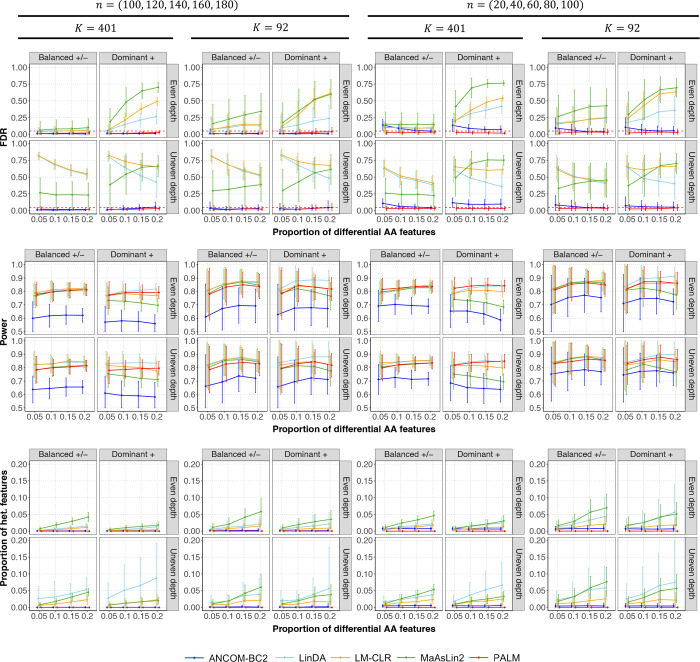
Comparison of different methods in the meta-analysis of five simulated studies with correlated samples. The figure is divided into three sections, each representing a benchmark metric (y-axis): empirical FDR (top; the horizontal line indicates the target FDR), power (middle), and the proportion of heterogeneous features (bottom). Heterogeneous features are defined as those with variation in effect sizes across studies exceeding what is expected by chance (Cochran’s Q test q-value < 0.1). The x-axis represents the proportion of differential absolute abundance (AA) features. The top title shows the two scenarios of sample sizes n in five studies. The second title shows the two scenarios of feature size K. The column facet title represents two effect direction scenarios: Balanced +/−, where differential AA features have an equal probability of exhibiting positive or negative effects, and Dominant +, where all differential AA features exhibit positive effects. The row facet title represents two sequencing depth scenarios: one with even sequencing depth and one with uneven sequencing depth between the comparison groups. Each panel displays the mean estimated metrics with ± standard errors (indicated by error bars) based on 100 simulation replicates.

Moving beyond studies with independent samples, we next evaluated the performance of methods in meta-analyzing studies with correlated samples (Fig. 2). For this evaluation, LM-CLR refers to the linear mixed model and DESeq2 is omitted because it cannot handle correlated samples. PALM effectively controlled the FDR, maintained high power, and exhibited no effect heterogeneity across studies. While the sensitivity score filter in ANCOM-BC2 effectively reduced FDR, it also lowered power. LinDA, LM-CLR, and MaAsLin2 exhibited FDR inflation and false effect heterogeneity across studies.

### Computation time

**Fig. 3:**
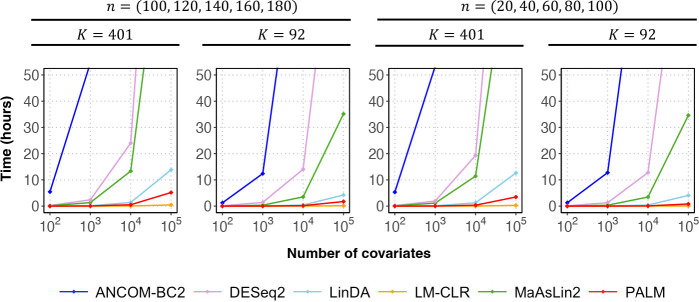
Comparison of computation time of different methods in the meta-analysis of five simulated studies with independent samples. The top title shows the two scenarios of sample sizes n in five studies. The second title shows the two scenarios of feature size K. Each plot shows the computation time in hours of different methods as a function of the number of covariates. The association scan was performed for one covariate at a time, and no parallel computing was used for all methods. Time beyond 50 hours is truncated. Values are averages over 100 simulation replicates for each setting.

We quantified the computation time of different methods in meta-analysis across varying sample sizes and feature sizes as the number of covariates increased from 10^2^ to 10^5^ (Fig. 3). LinDA, LM-CLR, and PALM demonstrated the highest computational efficiency, making them suitable for large-scale association scans. Increasing the number of features and covariates led to longer computation times for all methods, but the impact was minimal for LM-CLR and PALM. DESeq2 and MaAsLin2 exhibited moderate computational efficiency, and ANCOM-BC2 had the highest computational demand. Overall, all three methods were computationally impractical for association scans with more than 10^5^ covariates.

### Meta-analysis of microbiome association studies for colorectal cancer

We applied PALM to three real data applications. In the first application, we meta-analyzed five metagenomics studies^8,28–31^ to identify gut microbial features associated with colorectal cancer (CRC). These five studies were conducted on three continents and differed in sampling procedures, sample storage, and DNA extraction protocols. The combined sample size across studies was 574, comprising 284 CRC cases and 290 controls (Additional file 1: Table S1). All raw sequencing data were reprocessed using a unified bioinformatics pipeline for taxonomic profiling, as previously described^8^. The microbial community structures across studies showed significant differences (PERMANOVA test p-value < 10^−5^, Additional file 1: Fig. S2a). After filtering out microbial species with less than 20% prevalence across all samples, 401 species were retained for analysis.

**Fig. 4:**
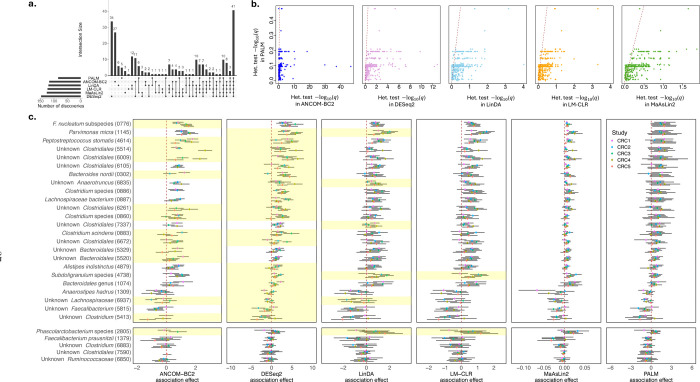
Results of the meta-analysis of microbiome association studies for colorectal cancer (CRC). **a**, The diagram shows the overlap of the CRC-associated species identified by different methods. **b**, Scatter plots of Cochran’s Q heterogeneity test −log_10_ (q-value) in the meta-analysis using summary statistics of PALM (y-axis) and those of ANCOM-BC2, DESeq2, LinDA, LM-CLR, and MaAsLin2 (x-axis) over all species (dots). **c**, Upper panel: x-axis represents a core set of CRC-associated species identified by all methods but exhibiting significant heterogeneity in effects across studies in at least one method. Each box shows the summary statistics of a method, including the association effect estimates across studies (color-coded dots) and their 95% confidence intervals (black lines). In the box for ANCOM-BC2, the effects of some studies were not shown, as they were removed by the sensitive score filter. The row for species with significant heterogeneous effects across studies is shaded yellow in each panel (Cochran’s Q test q-value < 0.1); Lower panel: similar format as the upper panel but for the five species exclusively identified by PALM.

ANCOM-BC2, DESeq2, LinDA, LM-CLR, MaAsLin2, and PALM identified 117, 147, 120, 123, 126, and 84 species, respectively (Fig. 4a, Additional file 2: Table S2). A core set of 41 species was consistently identified by all methods, including well-known species associated with CRC, such as *Fusobacterium nucleatum*, *Bacteroides fragilis*, and *Parvimonas micra* ^32,33^. Notably, the majority of PALM discoveries (79%) were also identified by at least three other methods. In contrast, ANCOM-BC2 and DESeq2 respectively identified 27 and 34 species that were not supported by other methods. Most of these species were very low-abundance, with average proportions below 5 × 10^−4^. Furthermore, many of the DESeq2 discoveries (64) and ANCOM-BC2 discoveries (59) failed to pass the sensitivity score filter when ANCOM-BC2 was applied to pooled-data analysis, suggesting they may be potential false signals.

PALM exclusively identified five species, including *Faecalibacterium prausnitzii*, species under *Phascolarctobacterium*, and unknown members of *Ruminococcaceae*, *Clostridiales*, and *Clostridium*. *Faecalibacterium prausnitzii* and certain members of *Ruminococcaceae* are well-recognized as protective microbes against CRC^31,34^. These microbes contribute to gut health by producing short-chain fatty acids and promoting anti-inflammatory pathways that help maintain gut barrier integrity and modulate immune responses^35,36^. Recent research has also suggested that *Faecalibacterium prausnitzii* may enhance the effectiveness of immunotherapy for CRC while mitigating its adverse effects^37^. For these species, PALM effect estimates were consistently negative across studies, whereas effect estimates from the other methods showed both negative and positive associations (Fig. 4c, lower panel).

The summary statistics from different methods exhibited varying levels of cross-study effect heterogeneity. PALM summary statistics showed no effect heterogeneity across all species, whereas other methods displayed heterogeneity in some species (Cochran’s Q test q-value < 0.1), with the strongest heterogeneity observed in ANCOM-BC2 and DESeq2 summary statistics (Fig. 4b, c). While it is impossible to know whether true effect sizes are heterogeneous in real data, our simulation results suggested that high observed heterogeneity may arise from inaccurate effect inference, even when the true effect sizes are homogeneous.

### Meta-analysis of microbiome studies linking to the metabolome

In the second application, we conducted a meta-analysis of eight microbiome-metabolome association studies^15,38–44^ to identify gut microbial features associated with gut metabolites. The total sample size across all studies was 2,127. The studies differed in microbiome sequencing platforms, metabolomic measurement methods, cohort demographics, and study designs (Additional file 1: Table S3). All raw sequencing data were reprocessed and metabolite identifiers were consolidated for uniformity, as described elsewhere^45^. The microbial community structures across studies showed significant differences (PERMANOVA test p-value < 10^−5^, Additional file 1: Fig. S2b). Most metabolites were quantified in only a subset of studies. To ensure better data quality, we focused on metabolites and genera with at least 10% prevalence within individual studies and excluded metabolites present in only one study. The final datasets for analysis contained 450 metabolites and 101 genera, yielding 43,722 metabolite-genus pairs for the association scan. Summary statistics for individual studies were generated by treating each metabolite as a covariate of interest and adjusting for disease status as a confounder.

**Fig. 5:**
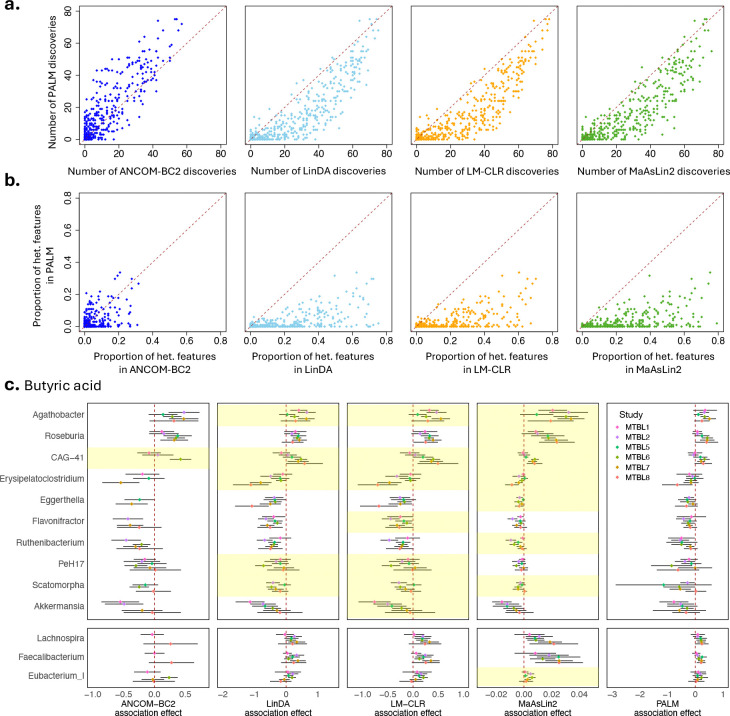
Results of the meta-analysis of microbiome-metabolome (MTBL) association studies. **a**, Scatter plots of the number of features identified by PALM (y-axis) and by ANCOM-BC2, LinDA, LM-CLR, and MaAsLin2 (x-axis) over all metabolites (dots). **b**, Scatter plots of proportions of features with significant cross-study effect heterogeneity (Cochran’s Q test q-value < 0.1) based on summary statistics of PALM (y-axis) and ANCOM-BC2, LinDA, LM-CLR, and MaAsLin2 (x-axis) over all metabolites (dots). **c**, Upper panel: x-axis represents a core set of butyric-acid-associated features identified by all methods but exhibiting significant heterogeneity in effects across studies in at least one method. Each box shows the summary statistics of a method, including the association effect estimates across studies (color-coded dots) and their 95% confidence intervals (black lines). In the box for ANCOM-BC2, the effects of some studies were not shown, as they were removed by the sensitive score filter. The row for features with significant heterogeneous effects across studies is shaded yellow in each panel (Cochran’s Q test q-value < 0.1); Lower panel: similar format as the upper panel but for the three well-known butyric-acid-associated features not included in the upper panel.

LinDA, LM-CLR, and MaAsLin2 identified significantly more features than PALM across metabolites (Fig. 5a, Additional file 3: Table S4). While ANCOM-BC2 identified a comparable number of features to PALM (Fig. 5a), the overlap between features identified by the two methods was low (Additional file 1: Fig. S7). In addition, we evaluated the number of associated metabolites across all features and characterized the top features associated with a large number of metabolites in the results of each method (Additional file 1: Fig. S8). Compared to other methods, the top features identified by PALM tend to be more abundant across individuals and are considered part of the human core microbiota^46,47^. We also found that this trend was driven by positive associations rather than negative associations (Additional file 1: Fig. S8b,c). Indeed, many of these features, such as *Phocaeicola*, *Bacteroides*, *Anaerostipes*, *Blautia*, *Faecalibacterium*, and *Roseburia*, are well-known producers of various metabolites, particularly short-chain fatty acids and other bioactive compounds essential for host health^48–50^.

We further investigated cross-study effect heterogeneity in the summary statistics generated by different methods. For almost all metabolites, LinDA, LM-CLR, and MaAsLin2 identified a larger proportion of features with heterogeneous effects compared to PALM (Fig. 5b). As an example, we highlighted the association results for butyric acid (Fig. 5c; additional results for cholic acid are shown in Additional file 1: Fig. S9). Many features associated with butyrate exhibited significant effect heterogeneity in the summary statistics produced by LinDA, LM-CLR, and MaAsLin2. The lower panel of Fig. 5c presents results for three well-known taxa contributing to butyrate production. These taxa were identified by LinDA, LM-CLR, MaAsLin2, and PALM but were missed by ANCOM-BC2. Their PALM effect estimates were consistently positive across studies, but the ANCOM-BC2 effect estimates had inconsistent directions.

### Analysis of microbiome and host genetics associations

In the third application, we conducted a gut microbiome and host genetics association study in a single infant cohort. The cohort consisted of 502 healthy term infants, with their gut microbiomes measured once during their first year of life. In our analysis, the microbial features included 109 amplicon sequence variants (ASVs) with a prevalence greater than 5%, and the covariates comprised 6,174,920 SNPs with a minor allele frequency (MAF) > 5%, resulting in about 6.7 × 10^8^ ASV-SNP pairs for the association scan. We applied computationally feasible methods LinDA, LM-CLR, and PALM to perform the mbGWAS. Several existing mbGWAS studies have also used LM-CLR in their analyses^25,51^. In all methods, we adjusted for age at enrollment, sex, the top five ancestry principal components, delivery mode, antibiotic exposure, and birth season as confounders.

**Fig. 6:**
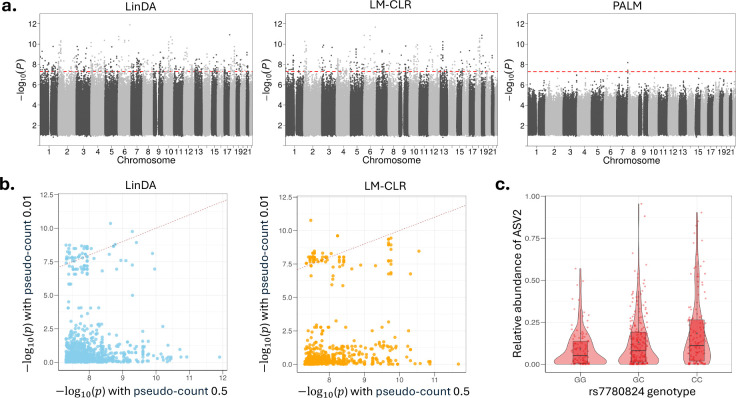
Results of the microbiome and host genetics GWAS. **a**, Manhattan plot aggregating the top associations with the microbial feature. Each SNP was tested against each of the 109 ASVs and the Manhattan plot shows the lowest resulting p-value for each SNP. The horizontal dashed line marks the genome-wide significance (p-value = 5 × 10^−8^). The top hit in PALM results involves feature ASV2 and SNP rs7780824. **b**, Comparison of p-values when using the pseudo-count of 0.5 (default) and 0.01 in LinDA and LM-CLR analysis. Each dot represents a significant ASV-SNP pair in the analysis using the default pseudo-count. **c**, Relative abundances of ASV2 stratified by groups of individuals based on the rs7780824 genotype. Data points are represented as dots. The boxplots show the median (center line), the first and third quartiles (box edges), and whiskers extending to 1.5 times the interquartile range. Violin plots illustrate the density distribution of the data points.

Our analyses were completed within 20 hours using 48 parallel cores on a 2.30 GHz Intel Xeon Gold 5118 processor. Using the genome-wide significance threshold (p-value < 5 × 10^−8^), LinDA identified 71 ASVs associated with at least one SNP, LM-CLR identified 50 ASVs, and PALM identified a single ASV (ASV2) associated with three SNPs (the top SNP is rs7780824 with p-value = 6.4 × 10^−9^, Fig. 6a). Many ASVs associated with a large number of SNPs in the LinDA and LM-CLR results tend to have low abundance (Additional file 1: Fig. S10a). Both methods rely on the CLR transformation, which requires adding a pseudo-count to handle zeros. To assess the impact of the pseudo-count, we re-ran LinDA and LM-CLR, varying the pseudo-count from the default value of 0.5 to 0.01. While both methods still identified many significant hits (Additional file 1: Fig. S10b), the majority differed from those identified previously (Fig. 6b), suggesting that the findings in LinDA and LM-CLR were unstable and may include false positives. The PALM-identified ASV2 belongs to genus *Escherichia-Shigella* and is highly prevalent in the cohort (92%), which naturally enhances its statistical power for detection in a moderate-sized GWAS. A clear trend of increased average relative abundance of ASV2 is observed in individuals with higher alternative allele dosages for rs7780824 (Fig. 6c).

## Discussion

The unique characteristics of microbiome data pose significant analytical challenges, which contribute to the poor replication of findings, particularly in large-scale association studies and meta-analyses. We introduce PALM, a statistical framework designed for fast and reliable association discovery. PALM directly models feature counts using quasi-Poisson regression to infer AA-level associations without requiring data preprocessing or distributional assumptions. By leveraging score statistics, PALM achieves computational efficiency and reliable association effect inference. Furthermore, PALM preserves homogeneous association signals across studies, even when microbial communities exhibit strong batch effects due to differences in technical batches, populations, study designs, and other factors.

We conducted comprehensive and realistic simulation studies to validate the performance of PALM in the meta-analysis of microbiome association studies. Recent research highlights that unrealistic simulations can compromise the fair benchmarking of microbiome differential abundance methods^52^, underscoring the importance of assessing the similarity between simulated and real data. In our simulations, we used five real microbiome datasets as templates to generate realistic multi-cohort microbiome datasets. The simulated data closely mirrored the structure of the real data (Additional file 1: Fig. S3), providing strong support for the reliability of our method comparisons. The simulations showed that PALM was the only method to consistently control FDR over diverse scenarios. It also demonstrated high power in identifying true covariate-associated features at the AA level. In our simulations, where the introduced association effects were truly homogeneous across studies, PALM was the only method to provide homogeneous association effect estimates over all scenarios. The performance of PALM in real data applications aligns with these simulation results. In the meta-analyses, PALM summary statistics exhibited lower cross-study heterogeneity compared to those of other methods. While PALM generally identified fewer microbial features than other methods, its discoveries were more stable and biologically meaningful.

To estimate the common compositional shift in RA effects, we use the median of the estimated RA effects. This estimator performs well in our simulations with up to 20% of differential AA features. We also examined a more challenging setting with 40% differential AA features (Additional file 1: Fig. S11). In this case, compositional bias is larger (especially when all effects are positive) and all methods show more severe FDR inflation; although PALM with the median compositional bias correction continues to yield lower empirical FDR than the other methods, it no longer controls FDR at the nominal level when all effects are positive. To address this, we propose an alternative estimator of the compositional effect (Additional file 1: Supplementary Methods 1.3). This approach adapts the idea in the microbiome meta-analysis method Melody^53^: we treat study-specific compositional biases as hyperparameters in a summary-statistics–based best-subset selection framework and jointly tune these correction factors across studies together with the subset-size hyperparameter. This approach is slower than using the median directly, and when the proportion of differential AA features is small, as in the main simulations, its effectiveness at correcting compositional bias is similar to that of the median (Additional file 1: Fig. S12).

The design of PALM score-statistic-based test enables it to efficiently handle association analyses involving a large number of features and covariates. Unlike many existing methods, it requires fitting a null model only once, regardless of the number of covariates, making it highly computationally scalable. This advantage was demonstrated in the mbGWAS application, where PALM efficiently analyzed millions of genetic variants. We also evaluated the Wald test under the PALM model. Although it attains higher power, it exhibits greater FDR inflation, particularly with small sample sizes (Additional file 1: Fig. S13). The score-statistic–based test demonstrates a more favorable power–FDR trade-off than the Wald test (Additional file 1: Fig. S14).

The current implementation of PALM is based on the quasi-Poisson model, which assumes a linear relationship between the variance and the mean. However, this mean-variance relationship may not hold for all microbial features. The PALM framework could be extended to incorporate the quasi-gamma-Poisson model, allowing the variance to follow a quadratic relationship with the mean. This article focuses on testing associations with one covariate at a time. Extending PALM to support multi-covariate association analyses would broaden its utility and application scope.

## Conclusions

High-throughput sequencing technology empowers researchers to profile microbiomes, yet the resulting data are highly complex, capturing only the noisy relative abundances of various microbial features. PALM addresses critical methodological gaps in modern large-scale microbiome association studies by integrating robust statistical modeling, computational scalability, and effective meta-analysis capabilities. By delivering reliable and generalizable results, PALM will accelerate discoveries with translational potential and advance microbiome research in health and disease. Beyond microbiome studies, PALM can be readily adapted to other types of high-dimensional compositional omics data, such as metabolomics and proteomics. We have provided an efficient R package for the broad utility of the method.

## Methods

### PALM framework

We first elucidate the relationship between RA and AA associations, laying the foundation for PALM’s approach to recovering AA associations. We then introduce the PALM framework in the context of meta-analysis.

#### Relationship between AA and RA associations

Consider n independent samples and K microbial features. For sample i, let $X_{i}$ be the covariate of interest, $N_{i}$ the sequencing depth, $Y_{ik}$ the read count assigned to feature $k,\;W_{ik}$ the (latent) AA of feature k in a unit volume, and $O_{i}=\sum_{k=1}^{K}W_{ik}$ the total microbial load. It is natural to model the mean of $W_{ik}$ as

(1) $$E\left(W_{ik}|X_{i}\right)=\exp\left(\beta_{0k}+\beta_{k}X_{i}\right),$$

where $\beta_{0k}$ is the intercept and $\beta_{k}$ is the AA-level association. Sequencing generates counts via a (feature-specific biased) multinomial sampling mechanism, leading to

(2) $$E\left(Y_{ik}|O_{i},X_{i}\right)=N_{i}b_{k}\frac{E\left(W_{ik}\;|\;O_{i},\;X_{i}\right)}{O_{i}}\approx N_{i}b_{k}\frac{E\left(W_{ik}\;|\;X_{i}\right)}{O_{i}},$$

where $b_{k}$ is a feature-level multiplicative factor accounting for measurement biases, such as differences in DNA extraction efficiency and primer binding/amplification efficiency in measuring various features^14^. In Additional file 1: Supplementary Methods 1.1, we show that the approximation $E\left(W_{ik}|O_{i},X_{i}\right)\approx E\left(W_{ik}|X_{i}\right)$ holds with typical microbiome data (large K, no overwhelmingly dominant feature, and no pervasive cross-feature dependence).

Since $O_{i}$ is the summation of $W_{ik}$’s, it will generally also be associated with $X_{i}$ whenever any $W_{ik}$ is associated with $X_{i}$. Therefore, we re-parameterize $O_{i}$ as $O_{i}=exp\left({\bar{O}}_{i}+\beta_{O}X_{i}\right)$, define the residual ${\bar{O}}_{i}:=logO_{i}-\beta_{O}X_{i}$, and express (2) as

(3) $$\begin{array}{c} E\left(Y_{ik}|O_{i},X_{i}\right)=\exp\left(\log N_{i}+\beta_{0k}^{*}+\beta_{k}^{*}X_{i}-{\bar{O}}_{i}\right),\\ \text{where}\;\beta_{0k}^{*}=\beta_{0k}+\log b_{k},\;\beta_{k}^{*}=\beta_{k}-\beta_{O}. \end{array}$$

Since $O_{i}$ is unobserved, we work with the marginal RA mean. We assume a mean-independence condition:

$$E\left(e^{-{\bar{O}}_{i}}|X_{i}\right)=C\left(\text{constant}\;\text{in}\;X_{i}\right).$$

Then the marginal RA mean can be expressed as

(4) $$\begin{array}{c} E\left(Y_{ik}|X_{i}\right)=N_{i}\exp\left(\beta_{0k}^{†}+\beta_{k}^{*}X_{i}\right),\\ \text{where}\;\beta_{0k}^{†}=\beta_{0k}^{*}+\log E\left(e^{-{\bar{O}}_{i}}|X_{i}\right). \end{array}$$

Thus, unidentifiable multiplicative factors ($b_{k}$ and the load factor $E(e^{-{\bar{O}}_{i}}|X_{i})$) are absorbed into the intercept $\beta_{0k}^{†}$, while the RA-level association $\beta_{k}^{*}$ equals the AA-level association shifted by the common quantity $\beta_{O}$ across all features.

#### Generating summary statistics for a study with independent samples

To estimate $\beta_{k}^{*}$, we propose using the quasi-Poisson regression with the RA mean specified in (4) and variance $Var\left(Y_{ik}\right)=\phi E\left(Y_{ik}\right)$, where ϕ is the dispersion parameter. This semi-parametric approach accounts for the overdispersion of count data and does not rely on specific distributional assumptions. Let $\theta_{k}={\left(\beta_{0k}^{†},\beta_{k}^{*}\right)}^{⊤},\;X_{i}={\left(1,X_{i}\right)}^{⊤}$, and $\mu_{ik}=N_{i}exp\left(X_{i}^{⊤}\theta_{k}\right)$. We employ the Firth bias-corrected^54^ Poisson quasi-score function

$$S\left(\theta_{k}\right)=\sum_{i=1}^{n}(Y_{ik}-\mu_{ik}+\frac{1}{2}h_{ik})\;X_{i}.$$

The $X_{i}$ can be expanded to include potential confounders. The $h_{ik}$ is the ith diagonal element of the ‘hat’ matrix $H=W^{1/2}X{\left(X^{⊤}WX\right)}^{-1}X^{⊤}W^{1/2}$, where the W is a diagonal matrix with diagonal elements $\left(\mu_{1k},\ldots ,\mu_{nk}\right)$ and X is the design matrix. The Firth correction effectively reduces bias in parameter estimates, particularly in cases of small sample sizes and features with a high proportion of zero counts^54^.

We can estimate $\beta_{k}^{*}$ by solving $S\left(\theta_{k}\right)=0$. However, fitting a separate model for each feature-covariate pair becomes computationally slow when the number of features and covariates is large. To address this, we propose estimating $\beta_{k}^{*}$ using the score statistic. Let ${\tilde{\theta }}_{k}$ denote the estimate of $\theta_{k}$ under the null model, which is independent of the covariate of interest. Partition the quasi-score function $S\left(\theta_{k}\right)$ into ($S_{1},\;S_{0}$), corresponding to the parameter of interest $\beta_{k}^{*}$ and the nuisance parameters, respectively. Similarly, partition the quasi-information matrix $-\frac{\partial S\left(\theta_{k}\right)}{\partial \theta_{k}}=\sum_{i=1}^{n}\mu_{ik}X_{i}X_{i}^{⊤}$ as $\left[\begin{array}{ll} {ℐ}_{11} & {𝓘}_{10}\\ {𝓘}_{01} & {𝓘}_{00} \end{array}\right]$. The RA-level association effect $\beta_{k}^{*}$ is estimated using the score statistic

$${\hat{\beta }}_{k}^{*}={ℐ}_{1}^{-1}\left({\tilde{\theta }}_{k}\right)S_{1}\left({\tilde{\theta }}_{k}\right),$$

where ${ℐ}_{1}={ℐ}_{11}-{𝓘}_{10}{𝓘}_{00}^{-1}{𝓘}_{01}$. This estimate is derived from the Taylor series expansion of the quasi-score function. Score-statistic-based association analysis is widely used in modern GWAS due to its greater statistical accuracy and numerical stability compared to Wald-statistic-based analysis^55,56^.

The variance of ${\hat{\beta }}_{k}^{*}$ can be robustly estimated using the sandwich formula^57^

$$V_{k}^{*}={ℐ}_{1}^{-1}\left({\tilde{\theta }}_{k}\right)(\sum_{i=1}^{n}U_{i}\left({\tilde{\theta }}_{k}\right)U_{i}^{⊤}\left({\tilde{\theta }}_{k}\right)){ℐ}_{1}^{-1}\left({\tilde{\theta }}_{k}\right),$$

where $U_{i}=S_{1,i}-{𝓘}_{10}{𝓘}_{00}^{-1}S_{0,i},S_{1,i}$ and $S_{0,i}$ are the ith summand of $S_{1}$ and $S_{0}$, respectively.

#### Generating summary statistics for a study with correlated samples

For studies with correlated samples (e.g., longitudinal data, repeated measurements, or family-based data), the PALM summary statistics can be obtained in a similar manner, with adjusted variance estimates to account for correlation among samples within a cluster. Suppose we have n clusters in the data. Let i index clusters (i=1,…,n) and t index correlated samples within a particular cluster $\left(t=1\ldots ,T_{i}\right)$. The notation for data is defined similarly to the independent sample case, but subscript i is replaced with *it* to index sample t within cluster i. The quasi-score function can be written as

$$S\left(\theta_{k}\right)=\sum_{i=1}^{n}\sum_{t=1}^{T_{i}}\left(Y_{itk}-\mu_{itk}+\frac{1}{2}h_{itk}\right)\;X_{it}.$$

Let ($S_{1},\;S_{0}$) be the partition of $S\left(\theta_{k}\right)$ corresponding to the parameter of interest $\beta_{k}^{*}$ and the nuisance parameters. We estimate ${\tilde{\theta }}_{k}$, construct ${\hat{\beta }}_{k}^{*}$, and calculate $\left[\begin{array}{ll} {ℐ}_{11} & {𝓘}_{10}\\ {𝓘}_{01} & {𝓘}_{11} \end{array}\right]$ and ${ℐ}_{1}$ using the same procedures described above. The sandwich estimator for the variance of ${\hat{\beta }}_{k}^{*}$ is adjusted to account for correlated samples as

$$V_{k}^{*}={ℐ}_{1}^{-1}\left({\tilde{\theta }}_{k}\right)\left(\sum_{i=1}^{n}\left(\sum_{t=1}^{T_{i}}U_{it}\left({\tilde{\theta }}_{k}\right)\right){\left(\sum_{t=1}^{T_{i}}U_{it}\left({\tilde{\theta }}_{k}\right)\right)}^{⊤}\right){ℐ}_{1}^{-1}\left({\tilde{\theta }}_{k}\right),$$

where $U_{it}=S_{1,it}-{𝓘}_{10}{𝓘}_{00}^{-1}S_{0,it},S_{1,it}$ and $S_{0,it}$ are the itth summand of $S_{1}$ and $S_{0}$, respectively.

#### Recovering AA-level summary statistics

We are interested in detecting AA-level associations ($H_{0}:\beta_{k}=0$). Given the relationship between AA- and RA-level association effects, $\beta_{k}^{*}=\beta_{k}-\beta_{O}$, it follows that $\beta_{O}$ represents the common compositional effect shared by all features (k=1,…,K). Without additional assumptions, $\beta_{O}$ is not estimable, and AA-level effects are unidentifiable from RA-level effects. Here, we leverage the high dimensionality of microbial features and assume that the majority of features have no AA-level effects (i.e., $\beta_{k}=0$ for most k). Under this sparse AA signal assumption, we propose estimating $-\beta_{O}$ using median ${\left\{{\hat{\beta }}_{k}^{*}\right\}}_{k}$ and obtaining the estimate of the AA-level effect as

$${\hat{\beta }}_{k}={\hat{\beta }}_{k}^{*}-median{\left\{{\hat{\beta }}_{k}^{*}\right\}}_{k}.$$

A key advantage of using the median is its robustness to extreme values and outliers.

The variance of ${\hat{\beta }}_{k}$ can be calibrated from $V_{k}^{*}$ by taking into account the variability of the median as (Additional file 1: Supplementary Methods 1.2)^58^

$$V_{k}\approx V_{k}^{*}+\frac{K}{4{\left(\sum_{k=1}^{K}\frac{1}{\sqrt{2\pi V_{k}^{*}}}\right)}^{2}}.$$

The ${\hat{\beta }}_{k}$ and $V_{k}$ can be conveniently shared as AA-level summary statistics across studies and subsequently combined to perform meta-analysis for association testing.

#### Meta-analysis

Suppose we want to meta-analyze L microbiome association studies. For study ℓ=1,…,L, we obtain AA-level association effect estimate ${\hat{\beta }}_{k\ell }$ and its variance $V_{k\ell }$ for each feature k. A feature may be unobserved or excluded from a given study due to its low prevalence in that study. We define ${ℒ}_{k}$ as the set of indices for studies where feature k is observed. Under the fixed-effect model, the inverse-variance weighted estimate of the overall effect is

$${\hat{\beta }}_{k,\mathrm{meta}}=\frac{\sum_{\ell \in {ℒ}_{k}}{\hat{\beta }}_{k\ell }/V_{k\ell }}{\sum_{\ell \in {ℒ}_{k}}1/V_{k\ell }}.$$

The variance of ${\hat{\beta }}_{k,\mathrm{meta}}$ is estimated as

$$V_{k,\mathrm{meta}}=\frac{1}{\sum_{\ell \in {ℒ}_{k}}1/V_{k\ell }}.$$

Finally, we construct the test statistic $Q_{k,\mathrm{meta}}={\hat{\beta }}_{k,\mathrm{meta}}^{2}/V_{k,\mathrm{meta}}$ and calculate the p-value using the chi-square distribution with one degree of freedom.

### Comparison of methods

We compared the performance of PALM with ANCOM-BC2, DESeq2, LinDA, LM-CLR, and MaAsLin2. In all meta-analyses, both in simulations and real data applications, summary statistics for each method were generated at the individual study level. A fixed-effect meta-analysis was then performed by combining these summary statistics for association testing. In the simulation studies and the first two real data applications, microbial features associated with the covariate of interest were identified using the BH procedure with a target FDR of 0.05. In the third real data application (mbGWAS), we followed the mbGWAS convention^6,59,60^ and reported genome-wide significance at p-value < 5 × 10^−8^. All analyses were conducted in R (v4.3.1). Feature prevalence filters were applied externally, and no additional prevalence filters were applied within each method’s function.

- ANCOM-BC2: we used the ancombc function in the package ANCOMBC (v2.2.2) with the specified argument prv_cut = 0. Summary statistics lfc_labels1 and se_labels1 were extracted from the output.
- DESeq2: we used the R package DESeq2 (v1.48.2) with default settings, except that the estimation of size factors was set to use ‘poscounts’, following^61^. Differential abundance test was performed with the function DESeq, which uses the nbinomWald test function as the default. Summary statistics log2FoldChange and lfcSE were extracted from the output.
- LinDA: we used the linda function in the package MicrobiomeStat (v1.2) with the specified arguments feature.dat.type = ‘count’. Summary statistics log2FoldChange and lfcSE were extracted from the output.
- LM-CLR: we first added a pseudo-count (default: 0.5) to each cell of the count matrix and then applied the centered log-ratio transformation. To analyze a study with independent samples, we used the lm function available through the base R distribution for each transformed feature variable as the response. To analyze a study with correlated samples, we used the lmer function in the package lme4 (v1.1–34). Summary statistics were then extracted from the coefficient table of the output.
- MaAsLin2: we used the Maaslin2 function in the package Maaslin2 (v1.14.1) with the specified arguments min_prevalence = 0, transform = ‘AST’. Summary statistics coef and stderr were extracted from the output.

For all methods, the fixed-effect meta-analysis was performed using the function rma in the package metafor (v4.2–0) with specified argument method=‘EE’. The Cochran’s Q heterogeneity test p-value was also extracted from the output.

### Simulation strategy

We simulated microbiome data by mimicking the structure of the real metagenomics data from five studies for CRC^8^ using the package MIDASim (v0.1.0)^26^. MIDASim is a fast simulator that reproduces the distributional and correlation structure of a user-supplied template microbiome data. It supports controlled shifts in sequencing depth and means of microbial features. We considered K=401 species with a prevalence of at least 20% across the five studies in the real data and K=92 genera derived by collapsing the species-level data. To learn the structure of species (or genus) counts in each study, we used the MIDASim.setup function in nonparametric mode. The function returned the mean proportion vector (μ) and additional parameters (e.g., correlation matrices) that define the structure of the template data. We simulated two equally sized groups of samples (X=1 and X=0) by introducing differential AA signals into the mean proportion vector between the groups. Specifically, we randomly selected a subset of features as “active” features. Half of these were chosen randomly from relatively abundant features, defined as having an average proportion of at least 10^−3^ in the original data, while the other half were randomly selected from the remaining features. The mean proportion vectors for the two groups were initialized as $\mu_{1}=\mu_{0}=\mu$. For active features with positive effects, we increased their proportions in $\mu_{1}$, and for active features with negative effects, we increased their proportions in $\mu_{0}$. The fold increase for each active feature was randomly sampled from Uniform(1, Δ). After adjusting for all active features, $\mu_{1}$ and $\mu_{0}$ were normalized so that their elements summed to 1, ensuring they represented valid proportions. Note that the proportions of all features were shifted after this step. However, the active features were considered true differential AA features, driven by changes in the underlying AA between the two groups, which in turn caused the shift in RA for all features. Finally, we consolidated all parameters, including $\mu_{1}$ and $\mu_{0}$, the specified sequencing depths, and the other parameters learned from the template data, using the MIDASim.modify function. We then generated samples for the two groups using the MIDASim function. This procedure was repeated for each study, resulting in realistic simulated datasets closely resembling the original data (Additional file 1: Fig. S3). The randomly selected active features and their associated fold changes during the spike-in procedure were consistent across studies, representing homogeneous association effects.

We considered a broad range of scenarios in our simulations: (1) Two settings for feature size: species-level (K=401) or genus-level (K=92) data; (2) Two settings for sample sizes over the five studies: large sample size (n=100,120,140,160,180) or small sample size (n=20,40,60,80,100); The factor Δ, which controls the size of the fold change in proportion, was set to 5 for the large sample size setting and 10 for the small sample size setting. (3) Two effect direction settings: balanced positive/negative effects (active features had equal probabilities of having positive/negative effects) or dominant positive effects (all active features had positive effects). (4) Two sequencing depth unevenness settings: even (sequencing depths of all samples were randomly drawn from the pool of sequencing depths in the real CRC data) or uneven (further doubled the sequencing depth for the samples in a randomly picked group); (5) A range of active feature proportions from 0.05 to 0.2. Combining all these factors resulted in 64 simulation settings, and we generated 100 simulated datasets for each setting.

We conducted another set of simulations to meta-analyze studies with correlated samples. First, we simulated independent samples as described above. Next, we assigned every two samples $Y_{i1}$ and $Y_{i2}$ from the same group to form cluster i. To introduce correlation, we simulated another independent sample $Y_{i}^{*}$ under the same group as $Y_{i1}$ and $Y_{i2}$. This independent sample was then mixed with $Y_{i1}$ and $Y_{i2}$ to create the correlated samples $Y_{i1}^{*}=\left(Y_{i}^{*}+Y_{i1}\right)/2$ and $Y_{i2}^{*}=\left(Y_{i}^{*}+Y_{i2}\right)/2$ for cluster i.

### Analyzed datasets

#### Colorectal cancer (CRC) studies:

The five studies include subjects from five different countries. The sample sizes of the studies are 109, 127, 120, 114, and 104 (Additional file 1: Table S1). All raw sequencing data across studies were reprocessed using the same bioinformatics pipeline for taxonomic profiling^8^. We removed two samples with an outlier sequencing depth of less than 2,000. Our analysis focused on K=401 species after removing those with a prevalence of less than 20% (prevalence was defined as the percentage of non-zero counts in all samples across studies).

#### Metabolome (MTBL) studies:

In all studies, the microbiome and metabolome were profiled from the same fecal samples. The datasets were curated and processed as part of an established effort^45^. We used the eight adult studies from this resource, with sample sizes of 96, 294, 240, 220, 444, 382, 287, and 164. These studies exhibit notable heterogeneity in study design, cohort demographics, and technical aspects of microbiome and metabolite measurements (Additional file 1: Table S3). Our analysis focused on 450 HMDB-annotated metabolites with study-specific prevalence ≥ 10% in at least two studies and 101 genera with study-specific prevalence ≥ 10% in at least one study. Following standard practice for normalizing skewed metabolite data, we applied a rank-based inverse normal transformation to the metabolite variables.

#### Host genetics study:

The study is based on a population-based birth cohort of term-born and healthy infants^62^. The host genetics and microbiome data were collected from a subset of the cohort with 502 infants aged between 2 and 242 days. Most of the infants were non-Hispanic White. Genotypes were determined using the Illumina Genome Diversity Array (GDA, 8v1.0) at the Center for Inherited Disease Research (CIDR) (Baltimore, MD). Individuals were removed if they met any of the following quality control (QC) criteria: genotype vs. self-reported sex mismatches, unexpected duplicate samples, > 10% missing genotypes, and autosomal heterozygosity exceeding five standard deviations (SD=0.8%) above or below the mean of 30.4%. SNPs meeting any of the following QC were excluded: > 10% missing genotypes, exact Hardy-Weinberg p-value < 5 × 10^−8^, or genotype concordance < 90%. A total of 1,785,122 directly genotyped SNPs remained following QC. Ancestry principal components were estimated from autosomal SNPs with MAF > 0.005 and LD r2 < 0.10, using the 1,000 Genomes dataset as the reference. Imputation was performed using the TopMed reference panel (r2 version 1.0.0) of >100,000 whole genome sequences (human genome build 38) and the TopMed Imputation Server (version 1.7.3). Specifically, Eagle (version 2.4) was used for genotype phasing and minimac4 (version 1.0.2) was used for genotype imputation. Genotype dosages for 6,174,920 biallelic SNPs with MAF > 5% and imputation INFO score > 0.9 were included in the analysis.

The details of microbiome sample collection, processing, and sequencing were previously reported^63^ and were briefly summarized here. For the characterization of the gut microbiome, we extracted bacterial DNA from the stool samples collected at enrollment using the DNeasy PowerSoil Kit (Qiagen). The libraries were then sequenced on an Illumina MiSeq with 2 × 250 base pair reads. The 16S rRNA sequences were processed using the dada2 package, grouping the sequences into ASVs and assigning taxonomy based on the SILVA reference database. Our analysis focused on 109 ASVs with a prevalence greater than 5%.

## Supplementary Material

Supplement 1

Supplementary Information:

Additional file 1: Supplementary Methods 1.1–1.3, Figs S1–S14, and Tables S1, S3.

Additional file 2: Tables S2.

Additional file 3: Tables S4.

## Data Availability

The data for the CRC studies were obtained from https://github.com/zellerlab/crc_meta^64^. The data for the MTBL studies were obtained from https://github.com/borenstein-lab/microbiome-metabolome-curated-data^65^. Individual-level genotype data for the INSPIRE birth cohort analyzed in this study are available through the NIH database of Genotypes and Phenotypes (dbGaP) under controlled access, accession phs004036.v1.p1^66^. PALM is implemented in the R package PALM, publicly available at https://github.com/ZjpWei/PALM_package^67^. The site also contains a complete set of manuals and instructions. The scripts generating reported results are deposited at https://github.com/ZjpWei/PALM^68^ and Zenodo https://doi.org/10.5281/zenodo.17547702^69^ under the GPL-3.0 license.

## References

[1] Lynch SV, Pedersen O. The Human Intestinal Microbiome in Health and Disease. N Engl J Med. 2016;375(24):2369–2379. 27974040 10.1056/NEJMra1600266

[2] Lee JY, Bays DJ, Savage HP, Bäumler AJ. The human gut microbiome in health and disease: time for a new chapter? Infect Immun. 2024;92(11):e00302–24. 39347570 10.1128/iai.00302-24 PMC11556149

[3] Franzosa EA, Hsu T, Sirota-Madi A, Shafquat A, Abu-Ali G, Morgan XC, Sequencing and beyond: integrating molecular’omics’ for microbial community profiling. Nat Rev Microbiol. 2015;13(6):360–372. 25915636 10.1038/nrmicro3451 PMC4800835

[4] Zhang X, Li L, Butcher J, Stintzi A, Figeys D. Advancing functional and translational microbiome research using meta-omics approaches. Microbiome. 2019;7(1):154. 31810497 10.1186/s40168-019-0767-6 PMC6898977

[5] Mallick H, Rahnavard A, McIver LJ, Ma S, Zhang Y, Nguyen LH, Multivariable association discovery in population-scale meta-omics studies. PLoS Comput Biol. 2021;17(11):e1009442. 34784344 10.1371/journal.pcbi.1009442 PMC8714082

[6] Kurilshikov A, Medina-Gomez C, Bacigalupe R, Radjabzadeh D, Wang J, Demirkan A, Large-scale association analyses identify host factors influencing human gut microbiome composition. Nat Genet. 2021;53(2):156–165. 33462485 10.1038/s41588-020-00763-1 PMC8515199

[7] Duvallet C, Gibbons SM, Gurry T, Irizarry RA, Alm EJ. Meta-analysis of gut microbiome studies identifies disease-specific and shared responses. Nat Commun. 2017;8(1):1784. 29209090 10.1038/s41467-017-01973-8 PMC5716994

[8] Wirbel J, Pyl PT, Kartal E, Zych K, Kashani A, Milanese A, Meta-analysis of fecal metagenomes reveals global microbial signatures that are specific for colorectal cancer. Nat Med. 2019;25:679–689. 30936547 10.1038/s41591-019-0406-6 PMC7984229

[9] Muller E, Algavi YM, Borenstein E. A meta-analysis study of the robustness and universality of gut microbiome-metabolome associations. Microbiome. 2021;9(1):1–18. 34641974 10.1186/s40168-021-01149-z PMC8507343

[10] Ma S, Shungin D, Mallick H, Schirmer M, Nguyen LH, Kolde R, Population structure discovery in meta-analyzed microbial communities and inflammatory bowel disease using MMUPHin. Genome Biol. 2022;23(1):1–31. 36192803 10.1186/s13059-022-02753-4 PMC9531436

[11] Sanna S, Kurilshikov A, van der Graaf A, Fu J, Zhernakova A. Challenges and future directions for studying effects of host genetics on the gut microbiome. Nat Genet. 2022;54(2):100–106. 35115688 10.1038/s41588-021-00983-z

[12] Gloor GB, Wu JR, Pawlowsky-Glahn V, Egozcue JJ. It’s all relative: analyzing microbiome data as compositions. Ann Epidemiol. 2016;26(5):322–329. 27143475 10.1016/j.annepidem.2016.03.003

[13] Gloor GB, Macklaim JM, Pawlowsky-Glahn V, Egozcue JJ. Microbiome datasets are compositional: and this is not optional. Front Microbiol. 2017;8:2224. 29187837 10.3389/fmicb.2017.02224 PMC5695134

[14] McLaren MR, Willis AD, Callahan BJ. Consistent and correctable bias in metagenomics sequencing experiments. Elife. 2019;8:e46923. 31502536 10.7554/eLife.46923 PMC6739870

[15] Lloyd-Price J, Arze C, Ananthakrishnan AN, Schirmer M, Avila-Pacheco J, Poon TW, Multi-omics of the gut microbial ecosystem in inflammatory bowel diseases. Nature. 2019;569(7758):655–662. 31142855 10.1038/s41586-019-1237-9 PMC6650278

[16] Wang Y, LêCao KA. Managing batch effects in microbiome data. Brief Bioinform. 2020;21(6):1954–1970. 31776547 10.1093/bib/bbz105

[17] Love MI, Huber W, Anders S. Moderated estimation of fold change and dispersion for RNA-seq data with DESeq2. Genome Biol. 2014;15(12):550. 25516281 10.1186/s13059-014-0550-8 PMC4302049

[18] Zhou H, He K, Chen J, Zhang X. LinDA: linear models for differential abundance analysis of microbiome compositional data. Genome Biol. 2022;23(1):1–23. 35421994 10.1186/s13059-022-02655-5 PMC9012043

[19] Lin H, Peddada SD. Multigroup analysis of compositions of microbiomes with covariate adjustments and repeated measures. Nat Methods. 2024;21(1):83–91. 38158428 10.1038/s41592-023-02092-7 PMC10776411

[20] Costea PI, Zeller G, Sunagawa S, Bork P. A fair comparison. Nat Methods. 2014;11(4):359–359. 10.1038/nmeth.2897 24681719

[21] Weiss S, Xu ZZ, Peddada S, Amir A, Bittinger K, Gonzalez A, Normalization and microbial differential abundance strategies depend upon data characteristics. Microbiome. 2017;5:1–18. 28253908 10.1186/s40168-017-0237-y PMC5335496

[22] Leek JT, Scharpf RB, Bravo HC, Simcha D, Langmead B, Johnson WE, Tackling the widespread and critical impact of batch effects in high-throughput data. Nat Rev Genet. 2010;11(10):733–739. 20838408 10.1038/nrg2825 PMC3880143

[23] Nygaard V, Rødland EA, Hovig E. Methods that remove batch effects while retaining group differences may lead to exaggerated confidence in downstream analyses. Biostatistics. 2016;17(1):29–39. 26272994 10.1093/biostatistics/kxv027 PMC4679072

[24] Hu Y, Satten GA, Hu YJ. LOCOM: A logistic regression model for testing differential abundance in compositional microbiome data with false discovery rate control. PNAS. 2022;119(30):e2122788119. 35867822 10.1073/pnas.2122788119 PMC9335309

[25] Qin Y, Havulinna AS, Liu Y, Jousilahti P, Ritchie SC, Tokolyi A, Combined effects of host genetics and diet on human gut microbiota and incident disease in a single population cohort. Nat Genet. 2022;54(2):134–142. 35115689 10.1038/s41588-021-00991-z PMC9883041

[26] He M, Zhao N, Satten GA. MIDASim: a fast and simple simulator for realistic microbiome data. Microbiome. 2024;12(1):135. 39039570 10.1186/s40168-024-01822-z PMC11264979

[27] Benjamini Y, Hochberg Y. Controlling the false discovery rate: a practical and powerful approach to multiple testing. J R Stat Soc Series B. 1995;57(1):289–300.

[28] Yu J, Feng Q, Wong SH, Zhang D, yi Liang Q, Qin Y, Metagenomic analysis of faecal microbiome as a tool towards targeted non-invasive biomarkers for colorectal cancer. Gut. 2017;66(1):70–78. 26408641 10.1136/gutjnl-2015-309800

[29] Vogtmann E, Hua X, Zeller G, Sunagawa S, Voigt AY, Hercog R, Colorectal cancer and the human gut microbiome: reproducibility with whole-genome shotgun sequencing. PloS One. 2016;11(5):e0155362. 27171425 10.1371/journal.pone.0155362 PMC4865240

[30] Feng Q, Liang S, Jia H, Stadlmayr A, Tang L, Lan Z, Gut microbiome development along the colorectal adenoma–carcinoma sequence. Nat Commun. 2015;6(1):6528. 25758642 10.1038/ncomms7528

[31] Zeller G, Tap J, Voigt AY, Sunagawa S, Kultima JR, Costea PI, Potential of fecal microbiota for early-stage detection of colorectal cancer. Mol Syst Biol. 2014;10(11):766. 25432777 10.15252/msb.20145645 PMC4299606

[32] Zhao L, Zhang X, Zhou Y, Fu K, Lau HCH, Chun TWY, Parvimonas micra promotes colorectal tumorigenesis and is associated with prognosis of colorectal cancer patients. Oncogene. 2022;41(36):4200–4210. 35882981 10.1038/s41388-022-02395-7 PMC9439953

[33] Wong SH, Yu J. Gut microbiota in colorectal cancer: mechanisms of action and clinical applications. Nat Rev Gastroenterol Hepatol. 2019;16(11):690–704. 31554963 10.1038/s41575-019-0209-8

[34] Lopez-Siles M, Duncan SH, Garcia-Gil LJ, Martinez-Medina M. Faecalibacterium prausnitzii: from microbiology to diagnostics and prognostics. ISME J. 2017;11(4):841–852. 28045459 10.1038/ismej.2016.176 PMC5364359

[35] Sokol H, Pigneur B, Watterlot L, Lakhdari O, Bermúdez-Humarán LG, Gratadoux JJ, Faecalibacterium prausnitzii is an anti-inflammatory commensal bacterium identified by gut microbiota analysis of Crohn disease patients. Proc Natl Acad Sci USA. 2008;105(43):16731–16736. 18936492 10.1073/pnas.0804812105 PMC2575488

[36] Louis P, Flint HJ. Diversity, metabolism and microbial ecology of butyrate-producing bacteria from the human large intestine. FEMS Microbiol Lett. 2009;294(1):1–8. 19222573 10.1111/j.1574-6968.2009.01514.x

[37] Gao Y, Xu P, Sun D, Jiang Y, Lin XL, Han T, Faecalibacterium prausnitzii abrogates intestinal toxicity and promotes tumor immunity to increase the efficacy of dual CTLA4 and PD-1 checkpoint blockade. Cancer Res. 2023;83(22):3710–3725. 37602831 10.1158/0008-5472.CAN-23-0605

[38] Erawijantari PP, Mizutani S, Shiroma H, Shiba S, Nakajima T, Sakamoto T, Influence of gastrectomy for gastric cancer treatment on faecal microbiome and metabolome profiles. Gut. 2020;69(8):1404–1415. 31953253 10.1136/gutjnl-2019-319188 PMC7398469

[39] Yachida S, Mizutani S, Shiroma H, Shiba S, Nakajima T, Sakamoto T, Metagenomic and metabolomic analyses reveal distinct stage-specific phenotypes of the gut microbiota in colorectal cancer. Nat Med. 2019;25(6):968–976. 31171880 10.1038/s41591-019-0458-7

[40] Kim M, Vogtmann E, Ahlquist DA, Devens ME, Kisiel JB, Taylor WR, Fecal metabolomic signatures in colorectal adenoma patients are associated with gut microbiota and early events of colorectal cancer pathogenesis. MBio. 2020;11(1):10–1128. 10.1128/mBio.03186-19 PMC7029137 32071266

[41] Franzosa EA, Sirota-Madi A, Avila-Pacheco J, Fornelos N, Haiser HJ, Reinker S, Gut microbiome structure and metabolic activity in inflammatory bowel disease. Nat Microbiol. 2019;4(2):293–305. 30531976 10.1038/s41564-018-0306-4 PMC6342642

[42] Mars RA, Yang Y, Ward T, Houtti M, Priya S, Lekatz HR, Longitudinal multi-omics reveals subset-specific mechanisms underlying irritable bowel syndrome. Cell. 2020;182(6):1460–1473. 32916129 10.1016/j.cell.2020.08.007 PMC8109273

[43] Wang X, Yang S, Li S, Zhao L, Hao Y, Qin J, Aberrant gut microbiota alters host metabolome and impacts renal failure in humans and rodents. Gut. 2020;69(12):2131–2142. 32241904 10.1136/gutjnl-2019-319766 PMC7677483

[44] Poyet M, Groussin M, Gibbons SM, Avila-Pacheco J, Jiang X, Kearney SM, A library of human gut bacterial isolates paired with longitudinal multiomics data enables mechanistic microbiome research. Nat Med. 2019;25(9):1442–1452. 31477907 10.1038/s41591-019-0559-3

[45] Muller E, Algavi YM, Borenstein E. The gut microbiome-metabolome dataset collection: a curated resource for integrative meta-analysis. npj Biofilms Microbiomes. 2022;8(1):79. 36243731 10.1038/s41522-022-00345-5 PMC9569371

[46] Trosvik P, de Muinck EJ. Ecology of bacteria in the human gastrointestinal tract—identification of keystone and foundation taxa. Microbiome. 2015;3:1–12. 26455879 10.1186/s40168-015-0107-4 PMC4601151

[47] Shetty SA, Hugenholtz F, Lahti L, Smidt H, de Vos WM. Intestinal microbiome landscaping: insight in community assemblage and implications for microbial modulation strategies. FEMS Microbiol Rev. 2017;41(2):182–199. 28364729 10.1093/femsre/fuw045 PMC5399919

[48] Wexler AG, Goodman AL. An insider’s perspective: Bacteroides as a window into the microbiome. Nat Rev Microbiol. 2017;15:304–316. 10.1038/nmicrobiol.2017.26 PMC5679392 28440278

[49] Duncan SH, Louis P, Flint HJ. Butyrate-producing bacteria from the human large intestine. Appl Environ Microbiol. 2002;68(11):5186–5190. 12324374 10.1128/AEM.68.10.5186-5190.2002 PMC126392

[50] Lück R, Deppenmeier U. Genetic tools for the redirection of the central carbon flow towards the production of lactate in the human gut bacterium Phocaeicola (Bacteroides) vulgatus. Appl Microbiol Biotechnol. 2022;106:1211–1225. 35080666 10.1007/s00253-022-11777-6 PMC8816746

[51] Wang W, Zhang Y, Zhang X, Li C, Yuan L, Zhang D, Heritability and recursive influence of host genetics on the rumen microbiota drive body weight variance in male Hu sheep lambs. Microbiome. 2023;11(1):197. 37644504 10.1186/s40168-023-01642-7 PMC10463499

[52] Wirbel J, Essex M, Forslund SK, Zeller G. A realistic benchmark for differential abundance testing and confounder adjustment in human microbiome studies. Genome Biol. 2024;25(1):247. 39322959 10.1186/s13059-024-03390-9 PMC11423519

[53] Wei Z, Chen G, Tang ZZ. Melody: meta-analysis of microbiome association studies for discovering generalizable microbial signatures. Genome Biol. 2025;26(1):245. 40826361 10.1186/s13059-025-03721-4 PMC12359909

[54] Firth D. Bias reduction of maximum likelihood estimates. Biometrika. 1993;80(1):27–38.

[55] Lin DY, Tang ZZ. A general framework for detecting disease associations with rare variants in sequencing studies. Am J Hum Genet. 2011;89(3):354–367. 21885029 10.1016/j.ajhg.2011.07.015 PMC3169821

[56] Tang ZZ, Lin DY. Meta-analysis for discovering rare-variant associations: statistical methods and software programs. Am J Hum Genet. 2015;97(1):35–53. 26094574 10.1016/j.ajhg.2015.05.001 PMC4571037

[57] Heyde CC. Quasi-likelihood and its application: a general approach to optimal parameter estimation. Springer; 1997.

[58] Van der Vaart AW. Asymptotic statistics. vol. 3. Cambridge University Press; 2000.

[59] Bonder MJ, Kurilshikov A, Tigchelaar EF, The effect of host genetics on the gutmicrobiome. Nat Genet. 2016;48(11):1407–1412. 27694959 10.1038/ng.3663

[60] Goodrich JK, Davenport ER, Beaumont M, Genetic determinants of the gut microbiome in UK Twins. Cell Host Microbe. 2016;19(5):731–743. 27173935 10.1016/j.chom.2016.04.017 PMC4915943

[61] Nearing JT, Douglas GM, Hayes MG, MacDonald J, Desai DK, Allward N, Microbiome differential abundance methods produce different results across 38 datasets. Nat Commun. 2022;13(1):342. 35039521 10.1038/s41467-022-28034-z PMC8763921

[62] Larkin EK, Gebretsadik T, Moore ML, Anderson LJ, Dupont WD, Chappell JD, Objectives, design and enrollment results from the Infant Susceptibility to Pulmonary Infections and Asthma Following RSV Exposure Study (INSPIRE). BMC Pulm Med. 2015;15(1):45. 26021723 10.1186/s12890-015-0040-0 PMC4506623

[63] Rosas-Salazar C, Shilts MH, Tang ZZ, Hong Q, Turi KN, Snyder BM, Exclusive breast-feeding, the early-life microbiome and immune response, and common childhood respiratory illnesses. J Allergy Clin Immunol. 2022;150(3):612–621. 35283139 10.1016/j.jaci.2022.02.023 PMC9463089

[64] . Wirbel J. Colorectal cancer meta-analysis. Github. 2019;https://github.com/zellerlab/crc_meta.

[65] Borenstein E. The curated gut microbiome metabolome data resource. Github. 2024;https://github.com/borenstein-lab/microbiome-metabolome-curated-data.

[66] Environmental influences on Child Health Outcomes (ECHO) Program, Children’s Respiratory and Environmental Workgroup (CREW). Omic Studies in Children’s Respiratory and Environmental Health. NIH database of Genotypes and Phenotypes (dbGaP); 2026. Study accession: phs004036.v1.p1 (controlled access). Available from: https://www.ncbi.nlm.nih.gov/projects/gap/cgi-bin/study.cgi?study_id=phs004036.v1.p1.

[67] Wei Z, Hong Q, Chen G, Hartert TV, Rosas-Salazar C, Das SR, PALM R package. Github. 2025;https://github.com/ZjpWei/PALM_package.

[68] Wei Z, Hong Q, Chen G, Hartert TV, Rosas-Salazar C, Das SR, Fast and reliable association discovery in large-scale microbiome studies and meta-analyses using PALM. Github. 2025;https://github.com/ZjpWei/PALM.

[69] Wei Z, Hong Q, Chen G, Hartert TV, Rosas-Salazar C, Das SR, Fast and reliable association discovery in large-scale microbiome studies and meta-analyses using PALM. Zenodo. 2025; 10.5281/zenodo.17547702.

